# Supplementary material for: Epigenetic profiling of growth plate chondrocytes sheds insight into regulatory genetic variation influencing height
Source: eLife. 2017 Dec 5;6:e29329. doi: 10.7554/eLife.29329 (PMC5716665; doi:10.7554/eLife.29329)
Supplement: Transparent reporting form [file elife-29329-transrepform.docx]

***eLife’s* transparent reporting form**

We encourage authors to provide detailed information *within their submission* to facilitate the interpretation and replication of experiments. If you have any questions, please contact us: [editorial@elifesciences.org](mailto:editorial@elifesciences.org).

**Sample-size estimation**

- You should state whether an appropriate sample size was computed when the study was being designed
- You should state the statistical method of sample size computation and any required assumptions
- If no explicit power analysis was used, you should describe how you decided what sample (replicate) size (number) to use

Please outline where this information can be found within the submission (e.g., page numbers or figure legends), or explain why this information doesn’t apply to your submission:

Sample size estimates do not apply to our study. Our study performed epigenetic profiling of mouse tissues, and compared the profiling data with existing mouse and human epigenetic and genetic data.

**Replicates**

- You should report how often each experiment was performed
- You should include a definition of biological versus technical replication
- The data obtained should be provided and sufficient information should be provided to indicate the number of independent biological and/or technical replicates
- If you encountered any outliers, you should describe how these were handled
- Criteria for exclusion/inclusion of data should be clearly stated
- High-throughput sequence data should be uploaded before submission, with a private link for reviewers provided (these are available from both GEO and ArrayExpress)

Please outline where this information can be found within the submission (e.g., page numbers or figure legends), or explain why this information doesn’t apply to your submission:

Each experiment (distal and proximal femur) were performed using 2 biological replicates. Each biological replicate was comprised of DNA isolated from the femurs of approximately 8 mice. We have described this on page 20, lines 442-462. For functional experiments at the *CHSY1* locus: luciferase reporter experiments, N = 4 biological replicates per concentration (page 29, lines 676-679); CRISPR-Cas9 deletion experiments, N = 3 biological replicates per deletion (page 27, lines 620-625); Allele-specific expression experiments, N = 5 biological replicates per variant (page 30, lines 697-700); HOXD13 over-expression studies, N = 3 biological replicates per concentration per cell type (page 29, lines 683-686).

**Statistical reporting**

- Statistical analysis methods should be described and justified
- Raw data should be presented in figures whenever informative to do so (typically when N per group is less than 10)
- For each experiment, you should identify the statistical tests used, exact values of N, definitions of center, methods of multiple test correction, and dispersion and precision measures (e.g., mean, median, SD, SEM, confidence intervals; and, for the major substantive results, a measure of effect size (e.g., Pearson's r, Cohen's d)
- Report exact p-values wherever possible alongside the summary statistics and 95% confidence intervals. These should be reported for all key questions and not only when the p-value is less than 0.05.

1) For analyses of the significance of overlap between two epigenetic datasets, we calculated p-values by comparing our observed overlap to a “null” distribution generated from a permutation-based approach. This is described on page 23, lines 519-524. P-values reported are empiric p-values from the permutations.

2) For enrichment of ATAC-seq peaks with height GWAS data, we use a permutation-based approach as built into the GoShifter software or using random GWAS loci to build a “null distribution.” The p-values reported are empiric p-values derived from comparing the observed overlap with the overlap from the permutations. These methods are described on page 23, lines 519-533.

3) For motif enrichment, p-values are calculated by comparing to a “null” distribution generated by sampling random GWAS variants. The p-values reported are empiric p-values derived from comparing the observed overlap with the overlap from the permutations. These methods are described on pages 24-25, lines 566-590.

4) For functional experiments in human cells using luciferase reporters, CRISPR-Cas9, or over-expression assays, p-values are calculated using un=paired two-sample Student’s T-test. These methods are described on page 29, lines 679-686, page 31, lines 724-728.

Exact values of N, definitions of center, methods of multiple test correction, and dispersion and precision measures do not apply to our paper.

Please outline where this information can be found within the submission (e.g., page numbers or figure legends), or explain why this information doesn’t apply to your submission:

(For large datasets, or papers with a very large number of statistical tests, you may upload a single table file with tests, Ns, etc., with reference to page numbers in the manuscript.)

**Additional data files (“source data”)**

- We encourage you to upload relevant additional data files, such as numerical data that are represented as a graph in a figure, or as a summary table
- Where provided, these should be in the most useful format, and they can be uploaded as “Source data” files linked to a main figure or table
- Include model definition files including the full list of parameters used
- Include code used for data analysis (e.g., R, MatLab)
- Avoid stating that data files are “available upon request”

Please indicate the figures or tables for which source data files have been provided:

We have provided the source data and corresponding R code for the histograms (new Figures 1-7, new figure-supplements 1-5) in a compressed file called Source Code.
